# Vibration Isolation and Noise Reduction Method Based on Phononic Crystal

**DOI:** 10.1155/2022/9903645

**Published:** 2022-10-10

**Authors:** Haiqing Li, Ping Sun

**Affiliations:** Liuzhou Vocational and Technical College, Liuzhou 545006, China

## Abstract

Phononic crystal is a new kind of sound insulation material, which has an elastic wave gap. When the elastic wave falls within the band gap, it will attenuate strongly in phononic crystal, and its attenuation degree is far greater than the predicted value of the mass density theorem. In this paper, the practical application of phononic crystals in low frequency sound insulation is taken as the breakthrough point. Firstly, the theory of phononic crystal band gap generation is calculated and analyzed, and the band structure of one-dimensional two-component Bragg scattering phononic crystals is calculated. Hypermesh is used to build the model, and the sound insulation performance of phononic crystals is simulated and analyzed through Nastran. A sound isolation test platform was built for the local resonant phononic crystal samples to verify its sound isolation ability.

## 1. Introduction

Vibration and noise reduction theory plays an important role in automobile, ship, aircraft, micromachinery, and other fields. It is also the basis of modern automobile comfort design, low-noise submarine design, aircraft instrument vibration isolation, micromachinery high-precision processing, and other high technology. In modern design and research, vibration and noise control of solid structures is a common concern [1–3]. The traditional theory of vibration and noise reduction has become mature after many years of development. It is urgent for the birth of new theories and methods to meet people's needs for low vibration and low-noise environment. Therefore, the concept of phononic crystal is proposed, and it is found that when an elastic wave propagates in periodic composite media, an elastic wave or acoustic band gap similar to a photon band gap will be generated, that is, the elastic wave or acoustic wave within the band gap frequency range will be prohibited to propagate in the medium.

In recent years, due to the bandgap characteristics of phononic crystals, which plays an important role in preventing the propagation of elastic waves, research studies on their practical application mostly focus on vibration control and noise prevention [4, 5]. Due to elastic wave propagation in the medium is the most direct impact vibration, by taking advantage of the band gap of phononic crystal characteristics can be used to reduce the spread of vibration energy. On the one hand, in mechanical manufacturing process can reduce the manufacturing error, improve the accuracy of the product, on the other hand can reduce the surrounding environment adverse effects of instruments and equipment, and improve its operating life [5–8]; as the propagation of noise obeys the elastic wave equation, phononic crystals also have the characteristics of bandgap, so this phenomenon can provide a new idea for the development of sound insulation materials.

## 2. Phononic Crystal Theory Method

Phononic crystals are periodic coincidence materials or structures with elastic wave band gaps and can be regarded as an extension of the crystal concept in solid state physics. In essence, the study of phononic crystals is to study elastic wave propagation in periodic inhomogeneous media [9, 10]. Therefore, the lattice and band theory in elastic dynamics and solid state physics is the theoretical basis of phononic crystals.

### 2.1. Elastodynamics

In elastodynamics, the stress-strain relationship should be clarified first, and the generalized Hooke's law should be understood. On this basis, the basic equations of elastodynamics can be established, and three basic equations describing the relationship between particle force, displacement, and stress-strain can be established [11–13]. The equation of motion with displacement as an unknown function is called the Wiener equation.

(1) $$\begin{array}{c} {\rho u}_{i}=\rho f_{i}+\overset{3}{\underset{j=1}{\sum }}\left\{\frac{\partial }{\partial x_{i}}\left(\lambda \frac{\partial u_{j}}{\partial x_{j}}\right)+\frac{\partial }{\partial x_{j}}\left[\mu \left(\frac{\partial u_{i}}{\partial x_{j}}+\frac{\partial u_{j}}{\partial x_{i}}\right)\right]\right\}\text{.} \end{array}$$

In the formula, *i*, *j*=1,2,3, *x*_1_, *x*_2_, *x*_3_ corresponds, respectively, to *x*, *y*, *z*, and *u*_1_, *u*_2_, *u*_3_ corresponds, respectively, to *u*_*x*_, *u*_*y*_, *u*_*z*_ or *u*, *v*, *w*. On the basis of this equation, the propagation characteristics of elastic waves in periodic composites are obtained. It provides a numerical calculation for the study of phononic crystals.

### 2.2. Lattice and Band Theory

The effect of phononic crystals on elastic waves is similar to that of atomic periodic potential fields on electrons. Due to this similarity, phononic crystal research directly adopts the concepts, description methods, and main conclusions related to lattice theory and band theory in solid physics [14–16].

The concept of the lattice is abstracted from the internal structure of a crystal. The lattice is translational periodicity and spatial symmetry. Because these two properties of crystal make the eigenfrequency and eigenmode of the field have a certain symmetry [17], the problem can be simplified, and the Bloch theorem is often used to explain the characteristics and laws of the eigenfield in crystal.

Energy band theory is the main theoretical basis for studying electron motion in a solid. It abstracts a solid into an ideal crystal with translational periodicity and symmetry, and simplifies the motion of the electron in solid to the motion of a single electron in a periodic potential field, thus establishing a series of methods to calculate the energy band of the electron in solid. Many basic physical properties of solids can be explained and explained in principle by the solid energy band theory [18–20]. Due to the phonon crystal analogy in crystals of the periodic structure, therefore, to calculate the solid band structure, usually select a Bloch functions form a complete, elastic wave function with the function of the phononic crystal base to expand, and then plug in the elastic dynamics equation, to determine the coefficient of expansion must satisfy the secular equation, the eigenvalue of energy are obtained accordingly.

### 2.3. Calculation Method of Band Gap Characteristics of Phononic Crystals

The study of the band gap mechanism and properties of phononic crystals depends on effective calculation methods of band gap properties. At present, the main calculation methods include the transfer matrix method, plane wave expansion method, finite difference time domain method, multiple scattering method, and concentrated mass method.

In this project, the transfer matrix method is used to calculate the band gap of one-dimensional phononic crystals. The transfer matrix method is based on the basic equation of continuous state parameters (stress, particle displacement, etc.), combined with the interface continuity condition, the transfer matrix of a single period is obtained. The corresponding dispersion relation and band structure are obtained by introducing periodic boundary conditions. At the same time, the finite period transmission characteristics can be obtained by multiplying finite transfer matrices.

According to the propagation law of SH wave and one-dimensional wave equation in phononic crystals, the relation between the NTH protocell and n-1 protocell of ideal phononic crystals can be obtained, *ψ*_*n*2_=*Tψ*_(*n* − 1)2_ where *T*=*K*_1_^−1^*H*_1_*H*_2_^−1^*K*_2_ is the transfer matrix [21]. Due to the periodicity of *x* direction, it can be obtained by using the Bloch theorem, *ψ*_*n*2_=*e*^*ika*^*ψ*_(*n* − 1)2_ where *k* is one-dimensional Bloch wave vector. The standard matrix eigenvalue problem can be obtained by combining the above two equations:

(2) $$\begin{array}{c} \left|T\left.-e^{ika}I\right|\right.=0\text{,} \end{array}$$

where *I* is the identity matrix.

By solving the eigenvalue of the matrix *T*, the dispersion relation between wave vector *K* and frequency *W* (perpendicular incidence) can be obtained.

(3) $$\begin{array}{c} \cos\;\left(ka\right)=\cos\;\left(\frac{w}{c_{1}}a_{1}\right)\cos\;\left(\frac{w}{c_{2}}a_{2}\right)-\frac{1}{2}\left(\frac{\rho_{1}c_{1}}{\rho_{2}c_{2}}+\frac{\rho_{2}c_{2}}{\rho_{1}c_{1}}\right)\sin\;\left(\frac{w}{c_{1}}a_{1}\right)\sin\left(\frac{w}{c_{2}}a_{2}\right)\text{,} \end{array}$$

where *a*=*a*_1_+*a*_2_are the lattice constant, and *ρ*_1_, *ρ*_2_, *c*_1_, *c*_2_ are the density and propagation speed of the two materials, respectively.

## 3. Calculation of Band Gap of One-Dimensional Phononic Crystals

**Figure 1:**
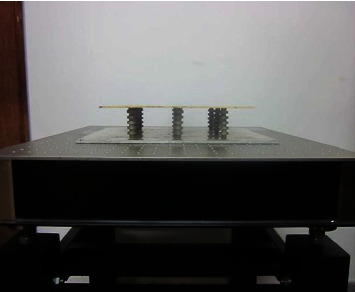
Phononic crystal composed of aluminum and epoxy resin.

In this study, phononic crystals composed of aluminum and epoxy resin mentioned in many literature were studied, and it was found that the band gaps were generally at a higher frequency. In order to explore the research method of phononic crystals, the band gap of ideal phononic crystals composed of these two materials was firstly calculated and analyzed [22, 23]. The specific structure is shown in Figure 1, and the materials are shown in Table 1. It is found that the band gap is at high frequency, the initial frequency of the first band gap is above several kilohertz, and the low frequency band gap is difficult to obtain.

Lattice periodic size *a* = 0.01 m, aluminum column diameter *d*_1_ = 0.020 m, period height *a*_1_ = 0.005 m; Silicon rubber diameter *d*_2_ = 0.012 m, *a*_2_ = 0.005 m. Specific material parameters are shown in Table 1.

To make the phononic crystals start at a low frequency, the density of the metallic part should be as high as possible, especially in applications where both the band gap start frequency and the band gap width are required. The change of elastic modulus within a certain range does not affect the band gap characteristics, so steel is selected for the metal part. As the rubber density increases, both the initial frequency and cutoff frequency of the band gap decrease, but as the density increases, the cutoff frequency of the band gap decreases more, and the width of the band gap decreases until the band gap disappears [24]. Therefore, the density of rubber should be appropriate, according to the rubber as silicone rubber.

Based on the above-given principles, material size parameters are determined: lattice periodic size *a*=0.05*m*, steel column diameter *d*_1_=0.028*m*, periodic height *a*_1_=0.03*m*; Silicone rubber diameter *d*_2_=0.025*m*, *a*_2_=0.02*m*. Specific material parameters are shown in Table 2.

**Figure 2:**
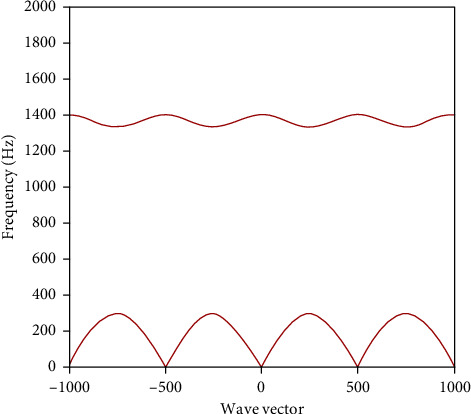
Band gap characteristics of one-dimensional ideal phononic crystals.

The band gap characteristics of one-dimensional ideal phononic crystals calculated by the transfer matrix method based on equivalent parameters are shown in Figure 2. It can be seen that the first gap is generated at 290 Hz∼1325 Hz.

## 4. Finite Element Simulation Verification

**Figure 3:**
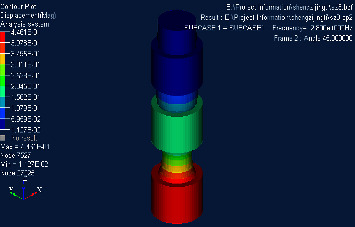
Cloud diagram of direct frequency response when frequency is 280 Hz.

**Figure 4:**
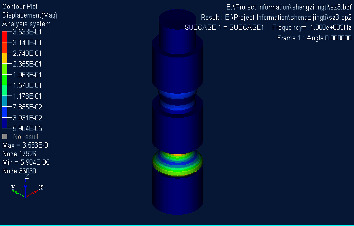
Cloud diagram of direct frequency response at 1360 Hz.

**Figure 5:**
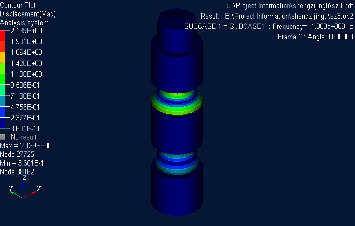
Cloud diagram of direct frequency response at 1380 Hz.

Based on the structure and parameters of the material mentioned above, the Hypermesh model was established and meshed. Nastran was used to calculate its direct frequency response and intercept part of the response cloud map, as shown in Figures 3–5. It can be concluded that the upper surface amplitude responses of phononic crystals are all less than 1 at 280 Hz∼1360 Hz, while the amplitude is greater than 1 at 1380 Hz. It can be judged that the band gap ranges from 280 Hz to 1360 Hz. Considering that the transfer matrix method results in infinite cycles and the finite element method calculates three cycles and the error of finite element numerical calculation, the results are considered to be credible [25].

## 5. Vibration Isolation and Noise Reduction Test Verification

### 5.1. Preparation of Phononic Crystals

**Figure 6:**
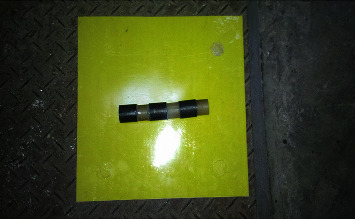
Phononic crystal is composed of steel and silicone rubber.

**Figure 7:**
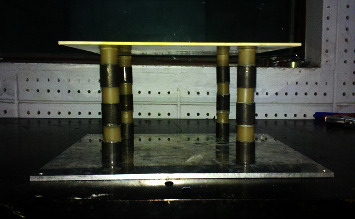
Array of four phononic crystals.

Based on the above-given calculation results and the material size verified by the finite element method, the structural design is carried out, and the two materials are periodically bonded together. Due to the limitation of cost and other conditions, only phononic crystals with three periods were made in this project. For ease of measurement and installation, four phononic crystals were made and arranged as shown in Figures 6 and 7.

### 5.2. Test Verification Method

Sensors used in the noise test are shown in Table 3. The sampling frequency is set to 12800 Hz. The sampling time was 3 s and the test times were 10 for each.

**Figure 8:**
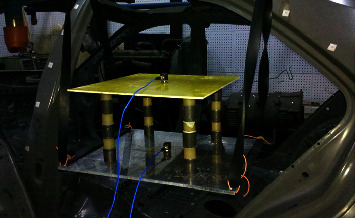
Suspension mode of phononic crystal.

**Figure 9:**
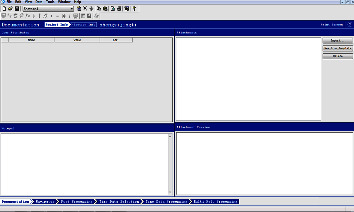
Test module in test software.

**Figure 10:**
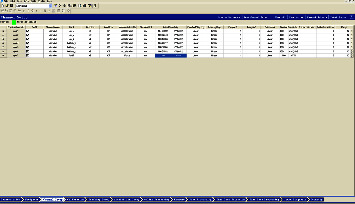
Channel Settings.

**Figure 11:**
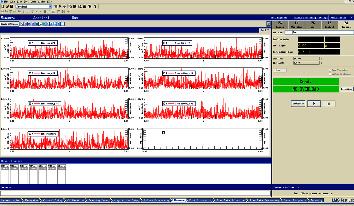
Setting and measurement interface.

1. Use rubber rope to suspend phonon crystal assembly on a suspension bracket of body in White, as shown in Figure 8
2. Install the three-way acceleration sensor, connect the sensor and computer to the signal acquisition front end, and start the test module of the test software, as shown in Figure 9
3. Channel Setup, as shown in Figure 10
4. Tracking Setup The test duration is 10 s, and the step length is 0.5 s
5. Acquisition Setup Set bandwidth and sampling frequency; See Figure 11
6. Start measuring

### 5.3. Effect and Analysis of Vibration Isolation and Noise Reduction

**Figure 12:**
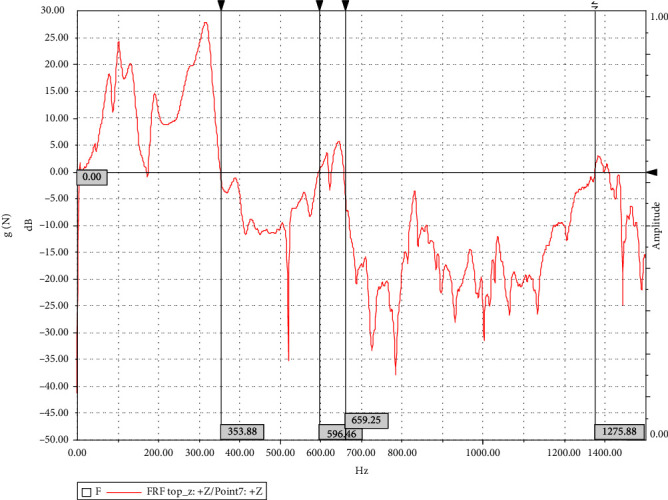
Frequency response function of the system.

Through the postprocessing function of the testing software, the frequency response function of the phonon crystal system is obtained, so as to clarify the band gap characteristics obtained by the test. The frequency response function obtained by processing is shown in Figure 12.

According to the frequency response function in Figure 12, the frequency response is less than 0 dB in the range of 353∼596 Hz and 659–1275 hz, that is, band gaps are generated in these frequency ranges. The band gap frequency range is partly consistent with the band gap obtained by theoretical calculation and simulation analysis, but the initial frequency is quite different [26, 27], around 60 Hz, and the band gap is discontinuous. Based on the analysis of the production process and test scheme, the main reasons for this difference may be as follows:

1. Due to the influence of manufacturing accuracy, there is a disalignment phenomenon between the two materials during bonding, which affects the vibration characteristics of phononic crystals;
2. As the upper and lower plates of the system have certain vibration characteristics, they contribute to the system during measurement. How to eliminate this contribution needs further research in the future;
3. It is better to use a shaking table to measure the frequency response function, but this test refers to the modal test method to suspend the system for testing, so there are certain test errors, and the test scheme needs to be further improved in the future research.

## 6. Conclusion

In order to explore the application of phonon crystal in the field of low frequency sound insulation, based on the low frequency band gap theory of phonon crystal, a one-dimensional two-element Bragg scattering phonon crystal is designed, its band structure is calculated, and its sound insulation ability is verified by experiments. The main research results are as follows:

1. The band results of the theoretical calculation method and finite element calculation are in good agreement with the experimental results, and the calculation method can be used for the band gap design of one-dimensional two-element Bragg scattering phonon crystal
2. The designed one-dimensional two-element Bragg scattering phonon crystal structure can form a low-frequency band gap and effectively isolate low-frequency vibration and noise

## Figures and Tables

**Table 1:** Material parameters.

Material	Density (*kg·m*^−3^)	Elasticity (10^10^*Pa*)	Poisson's ratio
Aluminum	2730	7.76	0.3
Epoxy resin	1180	0.435	0.4

**Table 2:** Material parameters.

Material	Density (*kg·m*^−3^)	Elasticity (10^10^*Pa*)	Poisson's ratio
Steel	7780	21.6	0.3
Silicone rubber	1481	4.2*e*–4	0.4

**Table 3:** Sensors used in noise test.

Sensor type	Microphone	Accelerometer
Model	PCB 378B02	PCB 356A02
Sensitivity	50 mV/Pa	10 mV/g
Frequency range	3.75 to 20000 Hz	1 to 20000 Hz
Mass loading	45.8 g	10.5 g

## Data Availability

If you need data support, please contact the contributor Haiqing Li, e-mail: lhq@lzzy.edu.cn.
